# A Dynamic Nomogram Predicting Portal Vein Thrombosis in Cirrhotic Patients During Primary Prophylaxis for Variceal Hemorrhage

**DOI:** 10.3389/fmed.2022.887995

**Published:** 2022-06-03

**Authors:** Shuo Zhang, Bing Ji, Xuan Zhong, Lan Zhong, Li Yang, Changqing Yang

**Affiliations:** ^1^Department of Gastroenterology and Hepatology, School of Medicine, Shanghai Tongji Hospital, Tongji University, Shanghai, China; ^2^Department of Gastroenterology, Shanghai East Hospital, Tongji University School of Medicine, Shanghai, China

**Keywords:** cirrhosis, portal hypertension, high-risk varices, portal vein thrombosis, nomogram

## Abstract

**Background:**

Portal vein thrombosis (PVT) would exert a further increase in resistance to portal blood flow, resulting in worsening portal hypertension and poor outcome. This study aimed to identify risk factors and develop an clinically applicable dynamic nomogram predicting the occurrence of PVT in cirrhotic patients during primary prophylaxis for variveal hemorrhage (VH).

**Methods:**

The multi-center retrospective study enrolled cirrhotic patients with high-risk varices, which were further divided into training and validation cohorts for 3 years follow-up. A dynamic nomogram based on the Cox proportional hazard regression model was developed with the cutoff value calculated by X-title analysis. The performance of the nomogram was evaluated with Harrell’s concordance index (C-index), calibration curve and decision curve analysis.

**Results:**

91 (34.0%) of the whole cohort were diagnosed with PVT during 3-year follow-up. Variables including carvedilol (*P* < 0.001), low portal vein velocity (*P* < 0.001), increased size of esophageal varices (*P* = 0.005), and high HbA1c (*P* < 0.001) and procalcitonin (*P* = 0.015) were identified to be independently associated with PVT, which were further incorporated into the dynamic nomogram with optimal cutoff (8.8 and 14.6) for risk-stratification. The C-indexes (0.894 of internal validation and 0.892 of external validation) and calibration curves demonstrated ideal discrimination and calibration. The thresholds for more reasonable application of the nomogram were 0–0.27, 0–0.66, and 0.04–1.00 at 1, 2, and 3-year, respectively.

**Conclusion:**

The dynamic nomogram could be accurately and reliably used for clinical risk-stratification of PVT in cirrhotic patients during primary prophylaxis for VH.

## Introduction

Portal vein thrombosis (PVT), causing occlusion of the portal vein with an annual incidence of approximately 10–15%, is a significant complication in liver cirrhosis (1, 2). Although PVT may often be asymptomatic, it is usually associated with severe clinical complications, including a higher risk of variceal hemorrhage (VH) and worse prognosis.

Doppler ultrasound (US) is the first-line imaging method to diagnose PVT, while its accuracy could be affected by technical limitations, including obesity, ascites, bowel gas, operator experience, and slow blood flow. Though circumventing the above situations, abdominal contrast-enhanced 4 phases (pre-contrast, arterial, portal, and late) computerized tomography (CECT) with radiation exposure and expensive nature could not be routinely applied as a short-term follow-up item. For cirrhotic patients underwent primary prophylaxis for VH, asymptomatic PVT is often failed to be timely diagnosed due to the relatively longer interval of follow up than those with VH history and the technical limitations of US and CECT. Besides, oral non-selective beta-blockers (NSBBs), one of approaches preventing VH, was suggested increasing the risk of PVT (2, 3). Studies had demonstrated that anticoagulant treatment could safely realize PVT recanalization and improve the prognosis of cirrhotic patients (4, 5), therefore emphasized the importance of prompt diagnosis of PVT in cirrhotic patients under primary prophylaxis for VH.

The multi-factorial origins of PVT, explained by Virchow’s triad, provides a favorable prospect for the application of combined predictive model (1, 2, 6). While, risk factors such as reduced portal vein blood flow, systemic inflammation, and acquired thrombophilia are yet clearly defined and permeated well in clinical practice (1). Recently, study based on cirrhotic patients demonstrated that low platelet count, portal vein velocity (PVV) < 15 cm/s and history of VH were factors independently associated with a high non-tumoral PVT risk (7). To our knowledge, a predictive scoring system focusing on patients who begin primary prophylaxis for VH has yet to be developed to evaluate the risk of PVT. An accurate predictive model is urgently required for risk-stratification guiding and clinical decision making.

Web-based dynamic nomogram, as a prediction tool, could be applied to quantify the likelihood of specific events of interest without inconvenient risk calculations of ordinary graphical nomogram (8). Therefore, the aim of this study was to determine the risk factors and further develop a dynamic nomogram predicting PVT in next 3 years during primary prophylaxis for VH.

## Patients and Methods

### Study Population

Patients underwent follow-up or newly diagnosed cirrhosis were routinely screened using upper gastrointestinal (GI) endoscopy, by which those identified high-risk esophageal varices (EV) needed to receive primary prophylaxis for VH. Consequently, these cirrhotic patients with clinically significant portal hypertension (CSPH) who began primary prophylaxis for VH were retrospectively enrolled from March 2016 to October 2018, of which patients followed the same inclusion and exclusion criteria from Shanghai Tongji Hospital and Shanghai East Hospital were set as training and validation cohorts, respectively.

Inclusion criteria included: (a) cirrhosis; (b) high-risk esophageal varices (EV) confirmed by upper gastrointestinal (GI) endoscopy; (c) partial and complete PVT screened out by both abdominal US and CECT. Imaging methods, including US, CECT, and magnetic resonance imaging (MRI) were applied to diagnose cirrhosis (9). Macro-structural changes included surface nodularity, widening of fissures, notching of the right lobe, and enlargement of the lateral segments of the left lobe and caudate lobe. Parenchymal changes included fibrotic septa and bridges and regenerative nodules. Signs of portal hypertension included splenomegaly, collateral venous circulation, and enlarged portal vein. For those failed to be determined cirrhosis, a value > 11.7 kPa by liver stiffness measurement (LSM) raised the suspicion of cirrhosis (10), and further serum markers and even liver biopsy were needed to make a diagnosis.

Exclusion criteria were as follows: (a) hemorrhage history; (b) extrahepatic malignancy or hepatocellular carcinoma (HCC); (c) ongoing infection or sepsis; (d) treated with anti-platelet or anti-coagulation medicine in the last 3 months; (e) splenectomy or partial splenic embolization; (f) lost follow-up. Baseline demographic and clinical data of patients were recorded at admission, during which the interval between laboratory tests and imaging examinations was no more than 72 h. This study had been approved by the ethical committee of Shanghai Tongji Hospital, following the Declaration of Helsinki.

### Diagnosis of the Risk of Gastrointestinal Bleeding

A standard upper GI endoscopic examination was performed by expert endoscopists, of which the findings were recorded in a standard format. The size of EV was graded as follows (11): small, < 30% of half esophageal lumen; medium, 30–60% of half esophageal lumen; large, > 60% of half esophageal lumen. According to the criteria proposed at the previous guidelines (12, 13), high-risk varices were defined as: (a) medium or large varices; (b) small varices with red signs or decompensated liver function.

### Abdominal Doppler Ultrasound and CECT

All measurements was detected using the same equipment with a 3.5-MHz transducer. Patients fasted for 8 h before examination. The operation and evaluation were performed by experienced sonographers, fixing the probe in a 30–60°angle between the Doppler beam and the portal vein’s long axis. PVV of the right branch was measured three times and traced at least 5 s each for calculating the average automatically.

The length of the spleen was evaluated as the maximum bipolar diameter passing through the splenic hilum.

PVT was defined as the partial or complete obstruction of the portal vein, the superior mesenteric vein, and the splenic vein using abdominal US or CECT examination, by which thrombosis appears as a low density, non-enhancing defect within the venous lumen (14).

### Primary Prophylaxis Against Variveal Hemorrhage

One of two approaches, including pharmacologic prophylaxis using carvedilol, or endoscopic prophylaxis using endoscopic variceal band ligation (EVL) was recommended for primary prophylaxis against VH (12, 13, 15), of which carvedilol is the recommended therapy for patients with high-risk small EV, and either NSBB or EVL is recommended for those with medium or large varices. Specific to our hospital, patients with small and medium EV were usually treated with oral carvedilol, while others with large varices or red color signs were commonly treated with EVL. Besides, drug tolerance and patients’ own preferences were also took into consideration of the treatment option. Patients who chose carvedilol therapy began at a dose of 6.25 mg once daily for 1 week, then increased to a maximum dose of 6.25 mg twice daily, and maintained as long as the systolic arterial pressure was not less than 90 mmHg (16). The details of the pre and post management and EVL technique followed previous description.

### Follow Up and End Point

Patients were routinely followed-up for every 3–6 months by serum examination, upper abdominal US and CECT. The primary end point was the occurrence of PVT during 3-year follow-up. When occurred acute VH, patients received EVL and short-term vasoactive agents during the acute phase and began carvedilol and EVL combined therapy at stability period.

### Statistical Analysis

The statistical analysis was performed using SPSS for Windows, version 22.0 (IBM SPSS Inc., Chicago, Ill, United States). Variable distributions were analyzed by Histogram and Shapiro-Wilk tests. Continuous variables expressed as mean ± standard deviation (SD) and median with interquartile range (IQR) were compared by Student’s unpaired *t*-test (a normal distribution) and Mann-Whitney *U*-test (a skewed distribution). Categorical variables showed as the absolute numbers with relative percentage by using chi-square test or Fisher’s exact test. In the training cohort, variables with statistically significant differences in univariate analysis were selected for further multivariate Cox proportional hazards regression analysis. Subsequently, factors with prognostic significance were utilized to build a dynamic nomogram using R studio software (version 4.1.1) for predicting the probability of PVT (17). The X-tile software (version 3.6.1) was used to determine the best cutoff value of nomogram (18). The Kaplan-Meier analysis based on the cutoff was further used to generate survival curves, and the log-rank test was used to evaluate statistical significance in the training and validation cohorts. To evaluate and validate the dynamic nomogram, training and validation cohorts were utilized for internal and external validations, respectively. The internal and external validations by using R studio software included Harrell’s concordance index (C-index), calibration curves with bootstrap resampling (1,000 resamples), receiver operating characteristic (ROC) curve with the areas under the receiver operating characteristic curve (AUROC) and decision curve analysis (DCA). Statistical analyses with two-sided *P*-value < 0.05 were considered significant.

## Results

### Demographics and Clinical Characteristics

**FIGURE 1:**
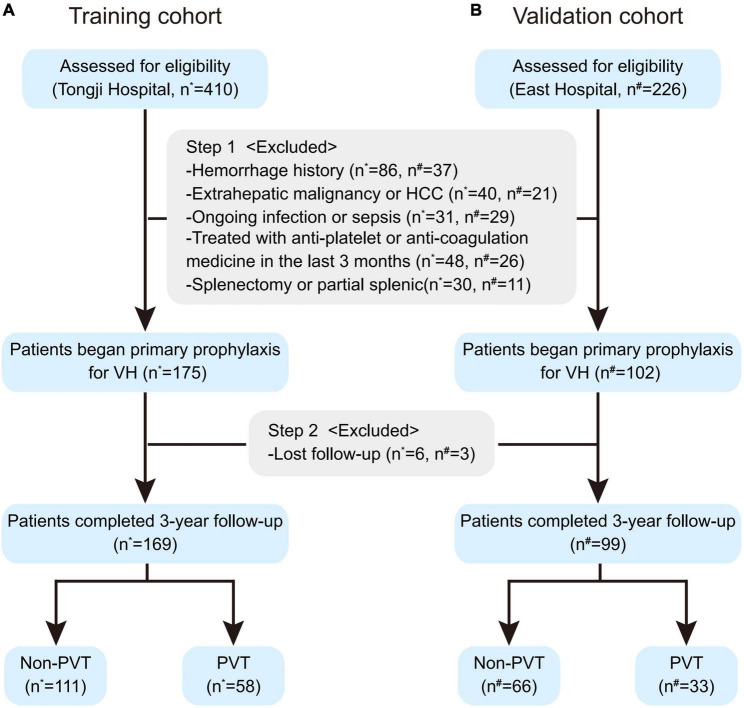
The flow chart of the training **(A)** and validation cohorts **(B)**. Patients assessed for eligibility were screened by the inclusion and exclusion criteria and started 3-year follow-up for primary prophylaxis against VH, during which some of these patients were further excluded due to lost follow-up. HCC, hepatocellular carcinoma; PVT, portal vein thrombosis; VH, variceal hemorrhage. *Patients enrolled from Shanghai Tongji Hospital. ^#^Patients enrolled from Shanghai East Hospital.

Over the study period, 636 cirrhotic patients with EV fulfilled the inclusion criteria, of which 359 patients were excluded for the following reasons (Figure 1): 123 (19.3%) patients with hemorrhage history; 61 (9.6%) with extrahepatic malignancy or HCC; 60 (9.4%) with ongoing infection or sepsis; 74 (11.6%) treated with anti-platelet or anti-coagulation medicine in the last 3 months; 41 (6.4%) splenectomy or partial splenic. 277 patients began primary prophylaxis started the 3-year follow-up, of which 9 (3.2%) lost follow-up were further excluded.

As shown in Table 1, a total of 268 patients were finally included in this study cohort, of which 26 (9.4%) occurred extrahepatic malignancy or HCC, 21 (7.5%) underwent splenectomy or partial splenic and 14 (5.2%) occurred acute VH during follow-up. There were no deaths among all these patients. For all these enrolled patients, 150 (56.0%) were male and the median age was 61.0 (IQR: 19.0) years. The predominant etiology of cirrhosis was hepatitis B virus (47.8%). 132 (49.3%) patients were Child-Turcotte-Pugh (CTP) B, 18 (6.7%) were CTP-C and the Model for End-Stage Liver Disease (MELD) value was 9.8 (IQR: 3.6). Over half of the patients had large EV (*n* = 153, 57.1%) and used carvedilol (*n* = 149, 55.6%) for the primary prophylaxis. 73 (27.2%) patients received prophylactic antibiotic therapy in hospitalization. There was no significant difference in characteristics between the training and validation cohorts (all *P*-value > 0.05). The characteristics at baseline classified with the occurrence of PVT were shown in Supplementary Table 1.

**TABLE 1:** Demographic and clinical characteristics of the training and validation cohorts.

Variables	Whole cohort (*n* = 268)	Training cohort (*n* = 169)	Validation cohort (*n* = 99)	*P*-value
Sex, male^§^	150 (56.0)	88 (52.1)	62 (62.6)	0.093
Age (year)^#^	61.0 (19.0)	61.0 (17.6)	62.0 (22.0)	0.141
Etiology of cirrhosis^§^				0.574
HBV	128 (47.8)	80 (47.3)	48 (48.5)	
HCV	26 (9.7)	20 (11.8)	6 (6.1)	
Alcohol	36 (13.4)	22 (13.0)	14 (14.1)	
Other	78 (29.0)	47 (27.8)	31 (31.3)	
CTP score^#^	7.0 (2.0)	7.0 (1.0)	7.0 (2.0)	0.199
CTP class^§^				0.207
A	118 (44.0)	78 (46.2)	40 (40.4)	
B	132 (49.3)	83 (49.1)	49 (49.5)	
C	18 (6.7)	8 (4.7)	10 (10.1)	
MELD^#^	9.8 (3.6)	9.7 (3.7)	9.9 (4.0)	0.188
Ascites^§^	148 (55.2)	95 (56.2)	53 (53.5)	0.670
PVT^§^	91 (34.0)	58 (34.3)	33 (33.3)	0.869
Size of EV^§^				0.797
Small	37 (13.8)	23 (13.6)	14 (14.1)	
Medium	78 (29.1)	47 (27.8)	31 (31.3)	
Large	153 (57.1)	99 (58.6)	54 (54.5)	
Therapy against VH^§^				0.603
EVL	119 (44.4)	73 (43.2)	46 (46.5)	
Carvedilol	149 (55.6)	96 (56.8)	53 (53.5)	
Antibiotic treatment^§^	73 (27.2)	48 (28.4)	25 (25.3)	0.576
PVV (cm/s)^#^	18.0 (6.0)	18.0 (6.0)	19.0 (6.0)	0.175
PVD (mm)^#^	12.0 (1.0)	12.0 (2.0)	12.0 (1.0)	0.807
Spleen length (mm)^#^	149.0 (15.8)	148.8 (12.9)	151.0 (19.0)	0.913
HbA1c (%)^#^	6.2 (1.4)	6.3 (1.2)	5.9 (1.9)	0.432
Leukocyte (× 10^9^/L)^#^	3.6 (3.2)	3.7 (3.6)	3.4 (2.7)	0.877
Platelet (× 10^9^/L)^#^	71.5 (57.3)	74.0 (59.0)	66 (55.0)	0.176
Neutrophil (%)^#^	62.2 (17.6)	63.6 (17.6)	60.8 (18.1)	0.877
CRP (mg/L)^#^	4.8 (6.1)	4.6 (5.4)	5.2 (6.8)	0.709
Procalcitonin (ng/mL)^#^	0.1 (0.1)	0.1 (0.1)	0.1 (0.2)	0.481
Albumin (g/L)*	32.8 ± 5.9	32.8 ± 8.2	32.9 ± 5.9	0.799
ALT (U/L)^#^	28.0 (18.0)	27.0 (18.0)	29.0 (17.0)	0.365
AST (U/L)^#^	35.0 (22.8)	35.0 (22.5)	34.0 (22.0)	0.848
TBIL (U/L)^#^	21.6 (16.1)	22.3 (15.3)	20.6 (17.2)	0.893
INR^#^	1.2 (0.2)	1.2 (0.2)	1.2 (0.3)	0.636

ALT, alanine aminotransferase; AST, aspartate aminotransferase; CRP, C-reactive protein; CTP, Child-Turcotte-Pugh; EV, esophageal varices; EVL, endoscopic variceal band ligation; HBV, hepatitis B virus; HCV, hepatitis C virus; INR, international normalized ratio; MELD, model for end-stage liver disease; PVD, portal vein diameter; PVT, portal vein thrombosis; PVV, portal vein velocity; Scr, serum creatinine; SMV, superior mesenteric vein; SV, splenic vein; TBIL, total bilirubin. *Mean ± standard deviation. ^#^Median (interquartile range, IQR). ^§^Number (percentage).

### Predictive Factors Associated With Portal Vein Thrombosis

91 (34.0%) patients completed follow-up were diagnosed with PVT, of whom 58 (21.6%) and 33 (12.3%) were in the training and validation cohorts, respectively. More specifically, 84 patients with PVT were determined by both US and CECT; 2 patients were determined by US alone; and the rest of 5 were determined by CECT alone. Details about the site of PVT were shown in Supplementary Table 1. In the training cohort, patients used carvedilol with low PVV, increased size of EV and high HbA1c, procalcitonin, leukocyte and ALT were comparatively at a higher risk occurring PVT (Table 2). Further multivariate Cox regression analysis identified carvedilol (OR = 4.134, *P* < 0.001), low PVV (OR = 0.704, *P* < 0.001), increased size of EV (OR = 2.027, *P* = 0.005), and high HbA1c (OR = 1.694, *P* < 0.001) and procalcitonin (OR = 3.516, *P* = 0.015) as independent risk factors for cirrhotic patients predicting PVT in next 3 years.

**TABLE 2:** Univariate and multivariate Cox regression analyses of the training cohort.

	Univariate analysis		Multivariate analysis	
Variables	Adjusted OR (95% CI)	*P*	Adjusted OR (95% CI)	*P*
CTP score	0.990 (0.813–1.207)	0.924		
MELD	1.065 (0.985–1.151)	0.113		
PVV (cm/s)	0.702 (0.646–0.764)	<0.001*	0.704 (0.635–0.781)	<0.001*
Spleen length (mm)	1.003 (0.993–1.013)	0.586		
Size of EV	1.643 (1.079–2.503)	0.021*	2.027 (1.242–3.308)	0.005*
Therapy against VH, carvedilol	3.259 (1.757–6.045)	<0.001*	4.134 (2.073–8.241)	<0.001*
Antibiotic treatment	1.083 (0.615–1.906)	0.783		
HbA1c (%)	1.640 (1.411–1.907)	<0.001*	1.694 (1.365–2.102)	<0.001*
Leukocyte (× 10^9^/L)	1.061 (1.003–1.121)	0.037*	1.015 (0.987–1.044)	0.284
Platelet (× 10^9^/L)	1.001 (0.999–1.003)	0.426		
CRP (mg/L)	1.005 (0.992–1.019)	0.449		
Procalcitonin (ng/mL)	4.256 (1.831–9.893)	0.001*	3.516 (1.274–9.699)	0.015*
ALT (U/L)	1.006 (1.002–1.011)	0.004*	1.002 (0.996–1.008)	0.575

CI, confidence interval; OR, odds ratio. *P < 0.05.

### Development of the Dynamic Nomogram

**FIGURE 2:**
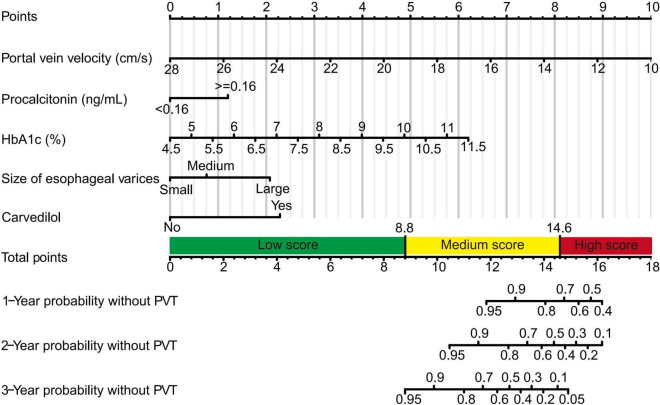
Nomogram predicting PVT in cirrhotic patients during primary prophylaxis for variceal hemorrhage. Five lines were firstly drawn upward to determine the points of the five predictors in the nomogram. The sum of these points was located on the “Total points” axis. Then, a line was drawn downward to determine the possibility of 1-, 2-, and 3-year probability without PVT.

Nomogram based on the multivariate Cox regression analysis was developed to estimate the 1, 2, and 3-year probability without PVT in cirrhotic patients during primary prophylaxis (Figure 2). In this model, vertical lines drawn from each variable axis corresponded with each variable’s points, the sum of which could be further converted into the probability without PVT diagnosis at the different time point. A practical online dynamic nomogram^1^ was further plotted to facilitate its global application (Supplementary Figure 1).

**FIGURE 3:**
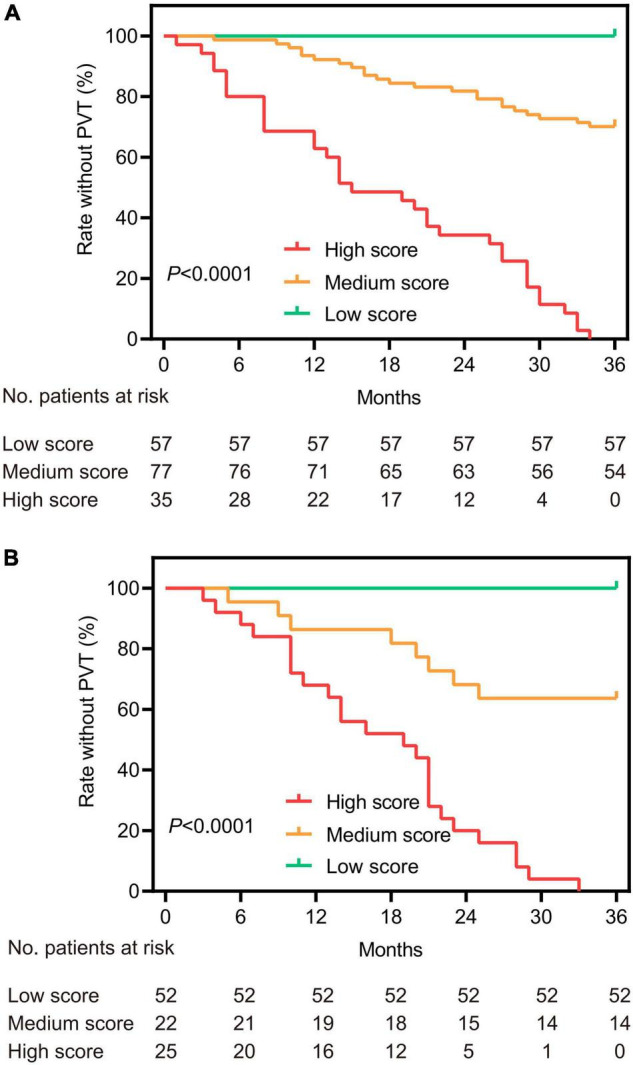
Kaplan–Meier survival curves based on the total points of the nomogram for the training **(A)** and validation **(B)** cohorts. According to the cutoff values of the nomogram, patients were divided into three categories and plotted the survival curves for the rate without PVT.

To avoid arbitrary cut point selection, X-tile program was applied to obtain the optimal cutoff values of the nomogram. In the training cohort, the total points of nomogram were calculated and further divided into low, medium and high scores based on the cutoff values of “8.8” and “14.6,” respectively (Figure 2), of which the 1, 2, and 3-year rate without PVT corresponding to the total points of 14.6 were 0.72, 0.46, and 0.10. Based on above cutoff values, the Kaplan–Meier curves for PVT were plotted and showed significant differences in both training (*P* < 0.0001, Figure 3A) and validation cohorts (*P* < 0.0001, Figure 3B).

### Evaluation and Validation of the Dynamic Nomogram

**FIGURE 4:**
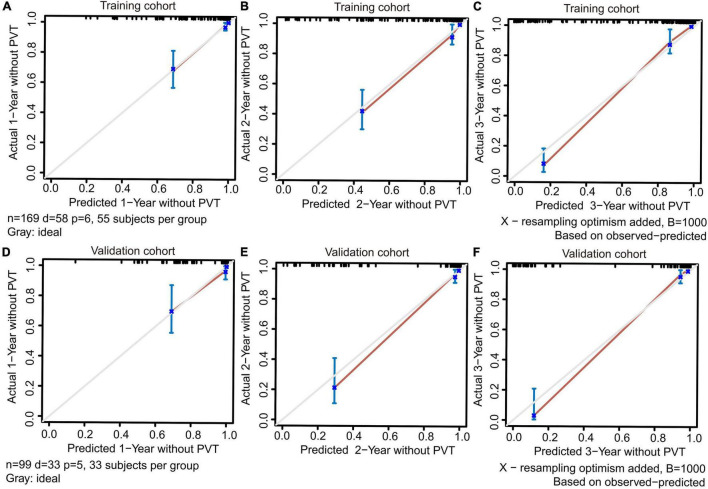
The calibration curves for predicting PVT at 1, 2, and 3-year in the training **(A–C)** and validation **(D–F)** cohorts. Perfect prediction would correspond to a slope of 1 (diagonal 45-degree gray line).

**FIGURE 5:**
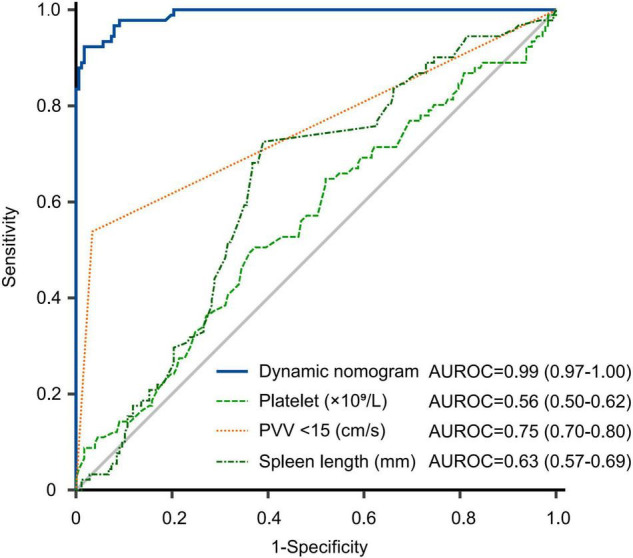
Receiver operating characteristic curve of nomogram and other factors for predicting PVT in the whole cohort. The predictive performance of the dynamic nomogram was comprartively better than platelet, PVV < 15 cm/s and spleen length. AUROC, area under receiver operating characteristic curve; PVV, portal vein velocity.

**FIGURE 6:**
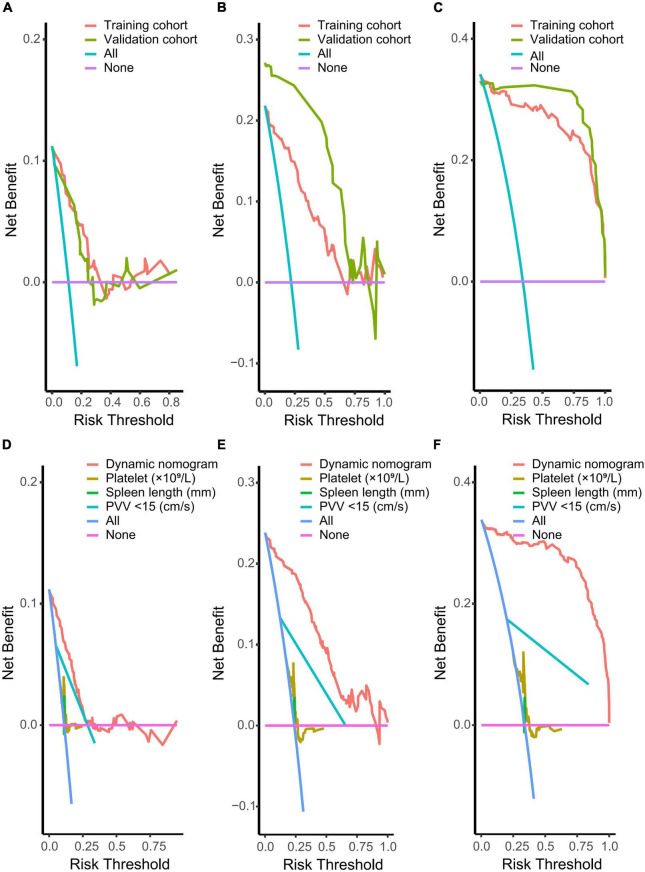
Decision curve analysis implicating the net benefit with respect to the use of the nomogram for predicting 1, 2, and 3-year PVT. **(A–C)** Decision curve analysis of nomogram for predicting PVT in both training and validation cohorts (*n* = 169 and *n* = 99, respectively). **(D–F)** Decision curve analysis of nomogram and other factors for predicting PVT in the whole cohort (*n* = 268). The *X*-axis represents the threshold probabilities, and the *Y*-axis measures the net benefit calculated by adding the true positives and subtracting the false positives. The horizontal line (None) along the *X*-axis assumed that PVT occurred in no patients, whereas the solid slant line (All) assumed that all patients will have PVT at a specific threshold probability. Other corresponding lines represented the net benefit of using the nomogram and other factors.

The training and validation cohorts were utilized for internal and external validations, evaluating the performance of the dynamic nomogram. The C-indexes were, respectively, 0.894 (95% CI = 0.831–0.957) and 0.892 (95% CI = 0.806–0.978) in the training and validation cohorts, showed a robust discrimination of the nomogram. Calibration curves testing the consistency showed that the PVT predicted probabilities agreed well with the observed probabilities in both training (Figures 4A–C) and validation (Figures 4D–F) cohorts. ROC curve demonstrated the diagnostic performance of the dynamic nomogram, with an AUROC of 0.99 (95% CI: 0.97–1.00) (Figure 5). DCA were performed to determine the optimal decision range of the nomogram (Figures 6A–C), of which the predicted risk thresholds were 0–0.27, 0–0.66, and 0.04–1.00 at 1, 2, and 3-year, respectively, and further confirmed the comparatively better clinical application of the nomogram than of other factors (Figures 6D–F).

## Discussion

PVT, a significant trigger of VH in cirrhotic patients, could not be identified until a routine follow-up, which may cause treatment delay and further increase the risk of hepatic decompensated and even life threat events. In this study, we identified that carvedilol, low PVV, increased size of EV, high HbA1c and procalcitonin could be used as risk factors and further constructed a predictive combined model of PVT for cirrhotic patients who began 3-year primary prophylaxis for VH, which was confirmed to provide continuous risk-stratification in the form of dynamic nomogram.

It is well established that venous stasis, hypercoagulability, and endothelial dysfunction are the pathophysiologic factors predisposing to thromboembolic events (1, 2, 6). PVV, decreased with the advancement of portal hypertension, is confirmed associated with the risk of PVT (19). A threshold PVV < 15 cm/s was described as the most predictive of PVT in both prospective and retrospective studies (7, 19, 20). Consistently, we identified that PVV is the independent risk factor of PVT, demonstrating portal vein hemodynamics as an important part evaluating the risk of PVT.

Consistent with previous studies, the size of EV, related to the stage of liver cirrhosis and portal vein pressure, was confirmed significantly associated with the risk of PVT (21, 22). For patients with high-risk varices, carvedilol, an NSBB with anti-α1 adrenergic, serves as a recommended therapy preventing the first VH, which can both decrease portal flow and act as a vasodilator of intrahepatic circulation. The relationship of carvedilol and PVT had been reported in previous studies (22, 23), of which Nery et al. (23) found that the effect of carvedilol on PVT development persisted after adjustment for resting heart rate and PVV. The potential mechanism of carvedilol for PVT development appears to be distinct from previous cognition. Given strong evidence for the benefits of carvedilol, cirrhotic patients should be closely followed up during pharmacologic prophylaxis for VH, rather than limited its use.

Hyperglycemia associated with inflammation and oxidative stress is an acknowledged risk of vascular dysfunction and cardiovascular disease in diabetes. In patients with cirrhosis, diabetes, increasing the risk of complications of cirrhosis, was proposed as a risk factor for PVT (24, 25). Although HbA1c was more closely associated with chronic complications than fasting plasma glucose (26), studies had not analyzed its effect on PVT. Specific to this study, patients with hemorrhage history and splenectomy affecting the level of HbA1c were excluded and further analysis identified that HbA1c had statistical significance for predicting PVT, which highlighted the importance of HbA1c monitoring and controlling in cirrhotic patients.

A growing body of data suggests that inflammation secondary to liver cirrhosis enhances both thrombin formation and hyperfibrinolysis in the portal system, thereby embolizing centrally and causing thrombosis (2, 27). Of note, the occurrence of PVT can in turn aggravate ischemic damage of enterocytes and liver, promoting subsequent bacterial translocation and accelerating the progression of the systemic inflammatory response (28). In the setting of advanced cirrhosis, the diagnositic performance of C-reactive protein (CRP) for endotoxemia is poor than that of procalcitonin (29). In this study, we identified that procalcitonin rather than CRP was a significant PVT predictor in cirrhotic patients began primary prophylaxis for VH. It might be explained that the combination of bacterial distribution and suppressed immunity in cirrhotic patients may lead to portal vein and systemic inflammation, which could further result in local or systemic vascular damage and initiate thrombus formation. Antibiotic treatment served as modulators of inflammation in cirrhosis could reduce circulating levels of gut-derived endotoxins (30) and theoretically reduce the risk of PVT. While, we identified that short-term antibiotic treatment during hospitalization followed serum examination had no significant effect on the development of PVT, which needed to be verified by further study.

This study focused on cirrhotic patients with high-risk varices who started primary prophylaxis for VH and identified carvedilol, low PVV, increased size of EV, and high HbA1c and procalcitonin to be risk factors for PVT. A dynamic nomogram based on above five variables were developed for risk-stratification according to the optimal cutoff values (8.8 and 14.6), which showed promising diagnostic performance (AUC = 0.99, 95% CI: 0.97–1.00), excellent discrimination (C-indexes = 0.894 of internal validation and 0.892 of external validation) and great calibration. DCA of the three different time points further determined the threshold more reliable predictions. According to the cutoff values of the nomogram, patients were divided into low, medium and high scores, of which the 1, 2, and 3-year rate without PVT corresponding to high score were less than 0.72, 0.46, and 0.10, respectively. In contrast, 3-year rate without PVT was nearly 0.95 in patients with low score of the nomogram. Therefore, patients should receive different follow up for PVT by using the dynamic nomogram. Meanwhile, we analyzed the factors of previous study (7) in this study population, which undoubtedly need to be validated by further prospective study. Firstly, these two studies did not aim to an exactly same patients group, which may partially explain the different results. Besides, this retrospective study failed to compare the effect of hemostatic factors on PVT. Regardless, This study could serve as one of explorations for precise management of cirrhotic patients who underwent primary prophylaxis against VH, which may provide novel insights for subsequent studies.

The limitations of this study were firstly caused by the comparatively small sample size and retrospective nature. Subgroups of the different sites and extent of PVT were not analyzed in the multivariate Cox regression model. Secondly, the positive pharmacological response of hepatic venous pressure gradient (HVPG) to carvedilol was not recorded to assess its effect on the development of PVT. Thirdly, the duration of follow-up was relatively short, which was hard to compare the longer-term prognosis of study cohort. There still needs a large, prospective study for further validation.

## Conclusion

In conclusion, we developed a web-based dynamic nomogram with reasonable accuracy for PVT in cirrhotic patients during primary prophylaxis for VH, which could be conveniently used for clinical decision making.

## Data Availability Statement

The raw data supporting the conclusions of this article will be made available by the authors, without undue reservation.

## Ethics Statement

The studies involving human participants were reviewed and approved by the Ethics Committee of Tongji Hospital. The patients/participants provided their written informed consent to participate in this study.

## Author Contributions

LY and CY conceived the study and supervised the work. SZ, BJ, XZ, and LZ collected the data and organized the statistical data. SZ drafted the manuscript. All authors contributed to the article and approved the submitted version.

## Conflict of Interest

The authors declare that the research was conducted in the absence of any commercial or financial relationships that could be construed as a potential conflict of interest.

## Publisher’s Note

All claims expressed in this article are solely those of the authors and do not necessarily represent those of their affiliated organizations, or those of the publisher, the editors and the reviewers. Any product that may be evaluated in this article, or claim that may be made by its manufacturer, is not guaranteed or endorsed by the publisher.

## Footnotes

1
https://nomogramshuozhang.shinyapps.io/Portal_vein_thrombosis/
