# Supplementary material for: A Dynamic Nomogram Predicting Portal Vein Thrombosis in Cirrhotic Patients During Primary Prophylaxis for Variceal Hemorrhage
Source: Front Med (Lausanne). 2022 Jun 3;9:887995. doi: 10.3389/fmed.2022.887995 (PMC9203843; doi:10.3389/fmed.2022.887995)
Supplement: Supplementary file 1 [file Data_Sheet_1.pdf]

Supplementary Material

Contents:

Supplementary Figures and legends:

Supplementary Figures	Title
**1**	The interface of the dynamic nomogram

Supplementary Tables:

Supplementary Tables	Title
**1**	Baseline characteristics of cirrhotic patients classified with the occurrence of PVT during 3-year follow-up

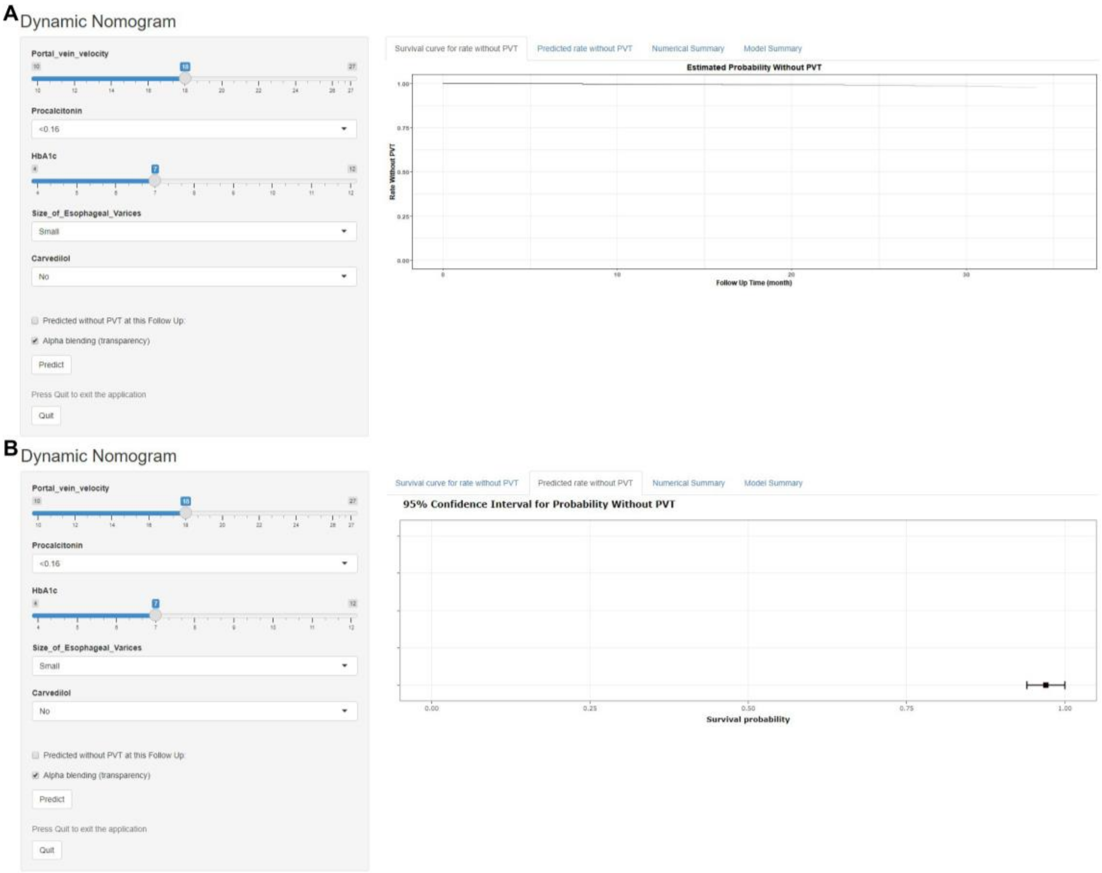

Supplementary Figure 1. The interface of the dynamic nomogram. **(A)** Survival plot for estimating the probability without PVT. The X-axis represents the follow up time (month), and the Y-axis measures the probability without PVT. **(B)** 95 % confidence interval for the probability without PVT. The X-axis represents the threshold probabilities without PVT.

Supplementary Table 2 Baseline characteristics of cirrhotic patients classified with the occurrence of PVT during 3-year follow-up.

**Variables**	**Patients with PVT (n=91)**	**Patients without PVT (n=177)**	***P*-value**
Sex, male§	47 (51.6)	103 (58.2)	0.307
Age (year)#	64.0 (22.0)	60.0 (17.0)	0.068
Etiology of cirrhosis§			0.001
HBV	60 (65.9)	68 (38.4)	
HCV	7 (7.7)	19 (10.7)	
Alcohol	7 (7.7)	29 (16.4)	
Autoimmune	6 (6.6)	38 (21.5)	
NAFLD	0 (0.0)	3 (1.7)	
Schistosomiasis	3 (3.3)	7 (4.0)	
Other	8 (8.8)	13 (7.3)	
CTP score#	7.0 (2.0)	7.0 (2.0)	0.071
CTP class§			0.150
A	34 (37.4)	84 (47.5)	
B	48 (52.7)	84 (47.5)	
C	9 (9.9)	9 (5.0)	
MELD#	10.3 (3.6)	9.5 (3.7)	0.070
Ascites§	57 (62.6)	91 (51.4)	0.080
Site of PVT§			
PV	57 (62.6)	0 (0)	
PV&SMV	18 (19.8)	0 (0)	
PV&SV	8 (8.8)	0 (0)	
PV&SMV&SV	8 (8.8)	0 (0)	
Size of EV§			<0.001
Small	4 (4.4)	33 (18.6)	
Medium	20 (22.0)	58 (32.8)	
Large	67 (73.6)	86 (48.6)	
Therapy against VH§			
EVL	16 (17.6)	103 (58.2)	
Carvedilol§	75 (82.4)	74 (41.8)	<0.001
PVV (cm/s)#	14.0 (3.0)	21.0 (5.0)	<0.001
PVD (mm)#	12.0 (2.0)	12.0 (1.0)	0.220
Spleen length (mm)#	154.0 (13.2)	148.8 (19.9)	<0.001
HbA1c (%)#	7.4 (1.3)	5.8 (0.8)	<0.001
Leukocyte (×10^9/L)#	3.6 (3.7)	3.7 (3.0)	0.794
Platelet (×10^9/L)#	63.0 (52.0)	74.0 (60.3)	0.134
Neutrophil (%)#	66.9 (18.2)	60.2 (17.3)	<0.001
CRP (mg/L)#	5.9 (5.7)	3.4 (5.6)	<0.001

Procalcitonin (ng/mL)#	0.2 (0.2)	0.1 (0.1)	<0.001
Albumin (g/L)*	31.8 ± 5.3	33.3 ± 6.1	0.053
ALT (U/L)#	28.0 (21.0)	27.0 (16.5)	0.980
AST (U/L)#	31.0 (16.0)	39.0 (19.8)	<0.001
TBIL (U/L)#	20.5 (13.9)	22.5 (15.9)	0.157
INR#	1.2 (0.2)	1.2 (0.2)	0.007
Scr (μmol/L)#	76.0 (30.0)	72.0 (24.0)	0.297

ALT, alanineaminotransferase; AST, aspartate aminotransferase; CRP, C-reactive protein; CTP, Child-Turcotte-Pugh; EV, esophageal varices; EVL, endoscopic variceal band ligation; HBV, hepatitis B virus; HCV, hepatitis C virus; INR, international normalized ratio; MELD, model for end-stage liver disease; NAFLD, nonalcoholic fatty liver disease; PV, portal vein; PVD, portal vein diameter; PVT, portal vein thrombosis; PVV, portal vein velocity; Scr, serum creatinine; SMV, superior mesenteric vein; SV, splenic vein; TBIL, total bilirubin.

* *Mean ± standard deviation.*

Median (interquartile range, IQR).

§ *Number (percentage).*
